# Obstetric and Neonatal Outcome of Pregnancy in Carney Complex: A Case Report

**DOI:** 10.3389/fendo.2020.00296

**Published:** 2020-05-08

**Authors:** Damian J. Ralser, Brigitte Strizek, Patrick Kupczyk, Birgit Stoffel-Wagner, Julia Altengarten, Andreas Müller, Joachim Woelfle, Ulrich Gembruch, Dietrich Klingmueller, Waltraut M. Merz, Anke Paschkowiak-Christes

**Affiliations:** ^1^Department of Obstetrics and Gynecology, University Bonn Medical School, Bonn, Germany; ^2^Department of Radiology, University Bonn Medical School, Bonn, Germany; ^3^Institute of Clinical Chemistry and Clinical Pharmacology, University Bonn Medical School, Bonn, Germany; ^4^Department of Dermatology, University Bonn Medical School, Bonn, Germany; ^5^Department of Neonatology and Pediatric Critical Care Medicine, University Bonn Medical School, Bonn, Germany; ^6^Pediatric Endocrinology and Diabetology Division, Children's Hospital, University Bonn Medical School, Bonn, Germany; ^7^Division of Endocrinology and Diabetes, Department of Medicine I, University Bonn Medical School, Bonn, Germany

**Keywords:** Carney complex, cushing syndrome, hypercortisolism, pregnancy, osteoporotic fracture, metyrapone, PPNAD, *PRKAR1A*

## Abstract

**Background:** Carney complex (CNC) is a rare multiple endocrine neoplasia syndrome with autosomal dominant inheritance. Affected individuals present with mucocutaneous lentigines/blue nevi, cardiac and noncardiac myxomatous tumors, and multiple endocrine tumors. Mutations in *PRKAR1A* have been identified as genetic cause of the disease. Here, we report on pregnancy, delivery and puerperium in a woman with genetically confirmed CNC and her newborn.

**Case:** The 31 year-old gravida 5 para 1 with CNC was referred at 26 weeks of gestation. Adrenocorticotropin-independent hypercortisolism, hyperglycemia, hypertension, low serum potassium, and osteoporotic fractures were present. Treatment with metyrapone, a reversible 11-beta-hydroxylase inhibitor, was initiated. The maternal condition improved, and a 5 weeks' pregnancy prolongation could be achieved. Elective repeat cesarean section was performed at 31 weeks of gestation for recurrent vaginal bleeding. The neonate developed transient hyponatremia necessitating hydrocortisone substitution for 2 weeks.

**Conclusion:** In our case, treatment of CNC-associated hypercortisolism in pregnancy with metyrapone was effective. Maternal side effects did not occur. The newborn presented with transient hypocortisolism most likely due to transplacental drug effect. Our case illustrates that the treatment of rare diseases in pregnancy represents a challenge requiring interdisciplinary team work.

## Introduction

Carney complex [CNC, Mendelian Inheritance in Man (MIM) 160980] is a rare autosomal dominant multiple endocrine neoplasia syndrome. Affected individuals present with mucocutaneous lentigines/blue nevi, cardiac and noncardiac myxomatous tumors, schwannomas, and multiple endocrine tumors (1, 2). Mutations in *PRKAR1A*, encoding the protein kinase A type I-alpha regulatory subunit have been identified in CNC by Kirschner et al. (3). Disease-causing mutations in *PRKAR1A* are found in >70% of patients diagnosed with CNC (4). A second gene locus has been mapped on chromosome 2p16 with the causative gene awaiting identification (5). A detailed list of diagnostic criteria and clinical manifestations of CNC has been reviewed elsewhere (4, 6–9).

Here, we report the course of pregnancy, delivery and puerperium in a woman and her newborn with confirmed maternal CNC, which was characterized by adrenocorticotropin (ACTH)-independent hypercortisolism, hypertension and osteoporosis-related fractures in the mother and transient hyponatremia in the newborn.

## Case Report

**Figure 1:**
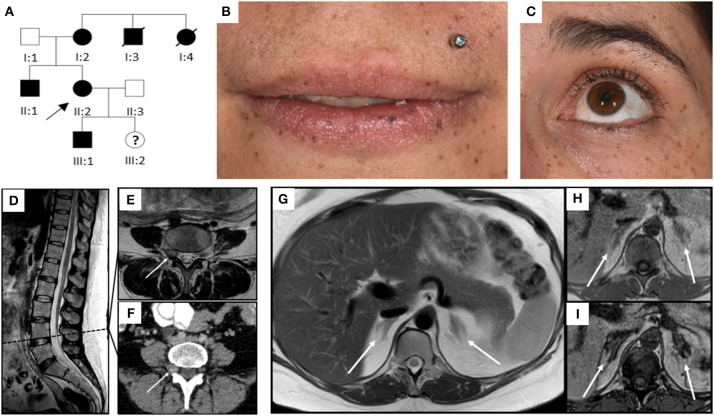
Pedigree, clinical appearance, and findings in magnetic resonance imaging. **(A)** Pedigree of the family with six affected individuals over three generations. Affected family members are shown in black; circles and squares denote females and males, respectively. The index patient is marked with an arrow (II:2). **(B,C)** Clinical appearance of II:2 with CNC-typical lentigines in the areas of **(B)** lip red, oral mucosa, **(C)** eyelids, conjunctiva, and eyelid margins. **(D–I)** Magnetic Resonance Imaging. **(D–F)** Sagittal T2 TSE of the lumbar spine **(D)**, axial T2 TSE **(E)** and post partum contrast enhanced CT **(F)** at the level of the intervertebral foramina L4. Small mass originating from the right spinal nerve root L4 (arrow) with inhomogeneous signal in T2w, most likely being a psammomatous melanotic schwannoma. As this was an incidental finding, T1w imaging was not performed. **(G–I)** Axial T2 TSE. **(G)**, axial chemical shift imaging with in phase **(H)** and opposed phase **(I)** at the level of the adrenal glands. Normal-sized adrenals without any masses (arrows). Besides, further criteria of PPNAD, such as hypointense (i.e., pigmented) foci in T1w and T2w and/or signal dropout in opposed phase, are not fulfilled.

A 31 year-old gravida 5 para 1 (II:2, Figure 1A) was referred to our department at 26 weeks of gestation with ACTH-independent hypercortisolism and suspected lumbar disc prolapse. She initially had presented at the referring hospital with severe headache and nausea. Preeclampsia had been ruled out. Further investigations had revealed elevated cortisol levels in both, serum and 24-h urinary collection. Serum ACTH levels were suppressed. A 24-h blood pressure profile had revealed hypertension. At the time of admission to our department, the patient reported severe movement-dependent pain in her left leg, a weight gain of 6 kilograms within the preceding 2 weeks (body mass index at the time of admission: 35.3 kg/m^2^), generalized edema, progressive muscular weakness, and visual deterioration. Physical examination revealed typical features of Cushing syndrome such as central obesity, cutis laxa, and striae distensae. Lentigines were present on her skin, including the areas of lip red, oral mucosa, eyelids, conjunctiva, and eyelid margins (Figures 1 B,C). Neurological findings were non-contributory. According to the antenatal records, blood pressure and weight gain had been within normal range during the first half of pregnancy. The patient and other family members had been diagnosed with Carney complex (CNC) after the patient's mother had undergone cardiac surgery for myocardial myxoma, see pedigree in Figure 1A; I:2. Molecular genetic diagnosis had revealed a large deletion within the *PRKAR1A* gene in all affected family members. Annual assessments recommended for CNC, including endocrine and cardiac investigations, had been taken up irregularly by our patient (8, 10), a pre-pregnancy hormonal status was therefore not available. Her obstetric history included one first-trimester miscarriage followed by one preterm delivery [elective cesarean section (CS) at 32 weeks of gestation for preeclampsia, with infection of the surgical site requiring operative revision]. Thereafter two first-trimester miscarriages occurred including one case of partial mole. CNC diagnosis had been established after the delivery.

Diagnostic workup in our department included laboratory tests, transthoracic echocardiography (TTE), ophthalmologic examination, and magnetic resonance imaging (MRI). ACTH-independent hypercortisolism was confirmed. The serum potassium level was slightly reduced, and blood glucose concentrations and homeostasis model assessment (HOMA) index were indicative of gestational diabetes. Laboratory findings are detailed in Table 1. TTE findings were normal, ophthalmologic evaluation revealed bilateral retinal edema. Serial blood pressure measurements showed hypertensive values. The MRI demonstrated a small mass originating from the right spinal nerve root L4, most likely corresponding to a psammomatous melanotic schwannoma, a typical finding with CNC (Figures 1D–F) (6, 7). Additionally, fractures of the right sacrum and left inferior pubic ramus were noted. No tumor was present within the adrenal glands, nor did the adrenal glands meet the criteria of primary pigmented nodular adrenal disease (PPNAD, Figures 1G–I) (11).

**Table 1:** Laboratory parameters at the time of hospital admission.

**Laboratory parameters**	**Value**	**Reference ranges**
Potassium (mmol/l)	3.01	3.5–5.1
Glucose (mmol/l)	78	74–109
Insulin (mU/l)	13.0	2.6–24.9
HOMA index	2.5	<2.5
TSH (μU/ml)	0.87	0.27–4.2
ACTH (pg/ml)	<1.5	7.2–63.3
Cortisol (μg/dl)	36.1	5–25
LH (U/l)	<0.3	1.0–95.6
FSH (mIU/ml)	<0.3	1.7–21.5
Estradiol (pg/ml)	2898.0	12–398
Prolactin (ng/ml)	49.5	4.79–23.3
DHEAS (μg/ml)	0.44	0.988–3.400
Urinary free cortisol (μg/d)	1,069	5–176

*HOMA, homeostasis model assessment; TSH, thyroid-stimulating hormone; LH, luteinizing hormone; FSH, follicle-stimulating hormone; DHEAS, dehydroepiandrosterone. Reference ranges are given for non-pregnant adults*.

An interdisciplinary team was set up for the patient's management. The aim was to treat maternal hypercortisolism, thereby improving the maternal condition, avoiding extreme prematurity, and decreasing perinatal risks (12). After extensive discussion with the patient, particularly regarding the lack of data on the safety of drug therapy in pregnancy, a joint decision for treatment with metyrapone (250 mg qid) was taken. Metyrapone causes reversible inhibition of 11-beta-hydroxylase, which consecutively inhibits cortisol biosynthesis (13, 14). It reduces cortisol levels, and was administered in one case report with CNC in pregnancy, and in another with ACTH-independent hypercortisolism in multiple pregnancy (13, 15, 16).

**Figure 2:**
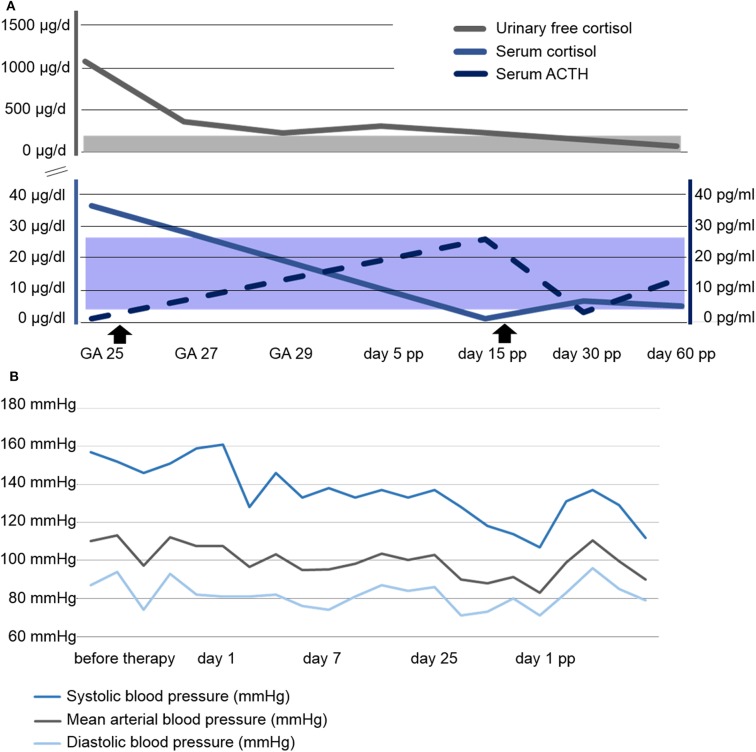
Course of laboratory parameters and blood pressure. **(A)** Course of serum and urinary free cortisol as well as serum ACTH concentrations. Shaded areas represent the respective normal ranges. The beginning and end of Metyrapone administration is marked with arrowsheads. **(B)** Course of blood pressure profile. GA, gestational age; pp, post partum.

Urinary free cortisol (UFC) concentrations rapidly normalized, see Figure 2A, and blood pressure remained within normal range on methyldopa 500 mg qid, see Figure 2B. A combined approach of analgesia (tramadol and paracetamol) and physiotherapy resolved the patient's pain and increased her mobility. Regression of the bilateral retinal edema resulted in improved vision. Glucose levels normalized after dietary advice. Potassium was supplemented orally. The generalized edema resolved, illustrated by a weight loss of 5 kilograms within the first 10 days of metyrapone treatment. Serial fetal ultrasound examinations revealed adequate fetal growth, with normal fetal anatomy and Doppler indices. From 29 weeks of gestation onwards, recurrent vaginal bleeding occurred. Therefore, at 31 weeks of gestation, the decision for delivery was taken, and elective repeat CS was performed. A preterm baby was delivered (female, 1740 grams, 73. centile, Apgar scores of 8/10/10 at 1, 5, and 10 min, respectively, umbilical cord arterial pH 7.33).

The newborn was transferred to the neonatal intensive care unit. Initially, the newborn's serum electrolyte levels were within normal range. Starting on the third day of life, the premature girl developed transient hyponatremia (minimal serum sodium concentration 112 mmol/l on day five) associated with an inadequately low serum cortisol level of 8.4 μg/dl, normal ACTH concentration of 27 pg/ml (reference range 7–63 pg/ml), raised renin concentration of 541 μU/ml (reference range 4.3–245 μU/ml), and an inadequately high sodium excretion (109 mmol/l). Hydrocortisone (HC) substitution was initiated with a dosage of 100 mg/m^2^/day, and rapidly tapered over a period of 14 days. Thereafter, the patient remained clinically well. Whereas no electrolyte disturbances were observed after discontinuation of HC substitution, renin concentration remained increased (1247 μU/ml).

The mother was discharged on post-operative day 5 on metyrapone. She was readmitted on day 10 with surgical site infection, and successfully treated with antibiotics. Interestingly, the ACTH concentration was now elevated, while cortisol serum levels were found to be reduced. Thus, metyrapone was tapered and HC substituted (25 mg daily). In total, the patient received metyrapone treatment for 47 days. During the further course, ACTH and cortisol levels were within normal range without medication. At the last follow-up, 6 weeks post partum, all symptoms had subsided.

A follow-up examination of the baby was performed 5 weeks after birth (corresponding to 36 weeks' of gestation). She presented clinically in good health. Laboratory investigations revealed normonatremia, raised ACTH (237 pg/ml) and persistently elevated—although decreasing—renin activity (217 μU/ml).

## Discussion

Hypercortisolism is a rare condition in pregnancy. Due to the overlap with pregnancy-associated changes like weight gain, edema, hyperglycemia and hypertension, diagnosis of hypercortisolism in pregnancy is challenging (15). However, hypercortisolism in pregnancy is associated with high maternal and fetal morbidity (17). Caimari et al. (12) reviewed 263 pregnancies with Cushing syndrome and found that maternal complications such as hypertension, gestational diabetes, preeclampsia, osteoporosis-related fractures, cardiac failure, wound infection, and psychiatric disorders were higher in untreated hypercortisolism. Perinatal morbidity included intrauterine growth restriction, prematurity, adrenal insufficiency, and intrauterine death (12, 15). Little is known about CNC in pregnancy. Our extensive literature search revealed only two published cases of pregnancy in patients with CNC (13, 18). The main findings comprise an increased cortisol synthesis, a worsening of hypertension, and a rise in fracture-related pain (13, 18). In one case, metyrapone was used for inhibition of cortisol synthesis. However, the medication was only administered for a few days because superimposed preeclampsia necessitated delivery (13).

Hypercortisolism in CNC is predominantly caused by primary pigmented nodular adrenocortical disease (PPNAD), which is present in 25–45% of all patients with CNC (6). Treatment consists of bilateral adrenalectomy (19). Even though MRI did not reveal signs of PPNAD in our patient we cannot exclude it as underlying cause of hypercortisolism since normal findings in MRI do not exclude PPNAD (11). Due to this uncertainty we decided against surgical treatment. For conservative management of hypercortisolism various drugs are available. These include adrenal enzyme inhibitors (ketoconazole, metyrapone, osilodrostat), adrenolytic agents (mitotane), and glucocorticoid-receptor antagonists (mifepristone). We decided to use the adrenal enzyme-inhibitor metyrapone, since most data on drug treatment of hypercortisolism in pregnancy are available for this drug. The majority of the other aforementioned drugs used in the treatment of hypercortisolism are contraindicated during pregnancy (12, 15, 20–22). A known side-effect of metyrapone is worsening hypertension and hypokalemia, which may develop secondary to accumulation of mineralocorticoid precursors. Interestingly, in our patient blood pressure was stable with antihypertensive treatment, and potassium levels were within normal range with oral supplementation.

Regarding the transient hyponatremia in the newborn, we assume that this phenomenon was caused by transplacental exposure to metyrapone. As in other forms of salt-wasting due to adrenal insufficiency, the manifestation of salt-loss in our neonatal patient did not present immediately after birth, but with a delay of 3 days. This delayed manifestation of adrenal insufficiency has been observed in a variety of conditions associated with adrenal insufficiency (most commonly congenital adrenal hyperplasia), with salt loss typically occurring between day 5 and 15 (23–25). This phenomenon has been explained by differences in an individual's homeostatic mechanism, including an increase of atrial natriuretic peptide or vasopressin hypersecretion. However, most of these mechanisms remain speculative (26).

Whereas the hyponatremia in our newborn patient was associated with an increased renin activity and increased urinary sodium excretion, surprisingly the ACTH concentration remained normal. We speculate that this lack of reactive ACTH increase in a newborn of 31 weeks' of gestation might be explained by a still immature hypothalamic-pituitary-adrenal axis, resulting in a blunted ACTH response. Clinically, we recommend a careful biochemical monitoring of newborns after intrauterine metyrapone exposure in order to avoid a delay in the diagnosis of drug-induced hypocortisolism and its sequelae.

We cannot exclude that the maternal hypercortisolism was pregnancy-associated in our patient, caused either by aberrant expression of adrenal LH/hCG receptors which consecutively cause an increase in cortisol synthesis during pregnancy (27) or stimulation of cortisol synthesis by placenta-related peptides such as placental corticotropin-releasing hormone (CRH) (28).

## Conclusion

CNC is a rare cause of hypercortisolism in pregnancy and should be considered in the presence of cushingoid clinical features and severe hypertension, combined with typical lentigines and a positive family history. Drug treatment with metyrapone is effective. Our case may serve as an example for the management of pregnancy, delivery, and pueperium in patients with CNC and their offspring, with a cautious monitoring of serum electrolytes in metyrapone-exposed newborns during the first week of life. Moreover, our case illustrates that the treatment of rare diseases in pregnancy is challenging and can only be achieved by interdisciplinary teamwork.

## Data Availability Statement

The datasets generated for this study are available on request to the corresponding author.

## Ethics Statement

Ethical review and approval was not required for the study on human participants in accordance with the local legislation and institutional requirements. The patient provided her written informed consent to participate in this study. Written informed consent was obtained from the individual for the publication of any potentially identifiable images or data included in this article.

## Author Contributions

All authors listed have made a substantial, direct and intellectual contribution to the work, and approved it for publication.

## Conflict of Interest

The authors declare that the research was conducted in the absence of any commercial or financial relationships that could be construed as a potential conflict of interest.

## Abbreviations

ACTH: adrenocorticotropin
CNC: Carney complex
CS: cesarean section
CRH: corticotropin-releasing hormone
DHEAS: dehydroepiandrosterone
FSH: follicle-stimulating hormone
GA: gestational age
GH: growth hormone
HOMA: homeostasis model assessment
HC: hydrocortisone
LCCSCT: large cell calcifying Sertoli cell tumor
LH: luteinizing hormone
MIM: Mendelian Inheritance in Man
MRI: magnetic resonance imaging
pp: post partum
PPNAD: primary pigmented nodular adrenal disease
*PRKAR1A*: protein kinase A regulatory subunit 1-alpha
TSH: thyroid-stimulating hormone
TTE: transthoracic echocardiography
UFC: urinary free cortisol.
